# Upcycling of Mixed Fruit Juice By‐Products for Flour Production and Application in Plant‐Based Burgers

**DOI:** 10.1111/1750-3841.71331

**Published:** 2026-08-06

**Authors:** Letícia da Cunha Espinosa, Tainara de Moraes Crizel, Paola de Souza, Giovanna Ribeiro Varesano, Aldrey Nathália Ribeiro Corrêa, Cecília Roratto Köhn, Adriano Brandelli, Simone Hickmann Flôres, Alessandro de Oliveira Rios

**Affiliations:** ^1^ Bioactive Compounds Laboratory Food Science and Technology Institute Federal University of Rio Grande do Sul Porto Alegre Rio Grande do Sul Brazil; ^2^ Laboratory of Nanotechnology and Applied Microbiology Food Science and Technology Institute Federal University of Rio Grande do Sul Porto Alegre Rio Grande do Sul Brazil

**Keywords:** agro‐industrial residues, dietary fiber, food waste valorization, meat analogues, plant‐based foods

## Abstract

Agro‐industrial by‐product valorization has emerged as an important strategy for developing sustainable functional ingredients aligned with circular economy principles. This study aimed to produce flour from mixed juice processing residues composed of strawberry, pineapple, and ginger and to evaluate its application in plant‐based burger formulations. The mixed fruit juice residue flour exhibited high total dietary fiber content (35.76 g/100 g), predominantly insoluble fiber, along with significant levels of phenolic compounds, resulting in elevated antioxidant capacity (ORAC: 377.21 µmol TE g^−^^1^) and antifungal activity, suggesting multifunctional and natural preservative properties. Plant‐based burgers were formulated with 10% and 15% mixed fruit juice residue flour incorporation and evaluated for physicochemical, technological, and sensory properties. Flour addition increased ash (1.70–2.95 and 3.36 g/100 g) and total fiber contents (7.19–8.28 and 8.99 g/100 g) while maintaining moisture levels comparable to similar plant‐based products. Technological analyses demonstrated suitable structural integrity, texture profile, and cooking performance, indicating that product quality was not impaired by flour incorporation. Sensory evaluation with 97 untrained panelists revealed high consumer acceptance, with five of seven attributes showing acceptance indices above 80% on a nine‐point hedonic scale. Overall acceptance scores were 7.56 and 7.40 for burgers containing 10% and 15% mixed fruit juice residue flour, respectively, demonstrating that incorporation did not compromise sensory perception despite fruit‐derived compounds. Overall, these results demonstrate that mixed fruit juice residues can be successfully converted into value‐added functional ingredients and incorporated into plant‐based meat analogs, contributing to food waste reduction, enhanced nutritional quality, and development of sustainable foods aligned with current consumer demands.

## 1 Introduction

The global food system is currently undergoing a significant transformation driven by increasing environmental concerns, population growth, and shifts in consumer dietary patterns. The growing demand for sustainable and nutritionally balanced foods has stimulated the development of plant‐based alternatives, particularly those intended to replace conventional meat products. Market projections indicate rapid expansion of the plant‐based food sector, with global revenue expected to increase substantially in the coming years, reflecting a structural shift toward more conscious and environmentally responsible consumption patterns (Research and Markets 2026). Moreover, global market revenue is expected to increase from USD 73.05 billion in 2026 to over USD 124.36 billion by 2030 (Research and Markets 2026).

Simultaneously, the food industry is facing increasing pressure to reduce waste generation and improve resource efficiency. Global fruit production has shown continuous growth in recent decades, reaching approximately 951 million tons in 2023, reflecting the expansion of demand for fresh fruits and fruit‐derived products (Statista, 2025). As a consequence, large volumes of agro‐industrial residues are generated during fruit and vegetable processing, many of which remain underutilized despite being rich sources of dietary fiber, phenolic compounds, and other bioactive constituents (Hasan et al. 2024). Previous research by our group demonstrated that fruit juice processing residues are rich in dietary fiber and bioactive compounds, highlighting their potential as functional ingredients in food applications (da Cunha Espinosa et al. 2026).

Dietary fiber intake is associated with several health benefits, including improved gastrointestinal function. Insoluble fibers contribute to increased fecal bulk, while soluble fibers enhance water retention and promote the production of beneficial metabolites, acting as prebiotics (von Muhlenbrock et al. 2025). In addition, high‐fiber diets have been linked to improvements in gut microbiota and reductions in inflammation. Fiber intake has also been associated with reduced risk of chronic diseases, including cardiovascular disorders. For instance, Da Silva et al. (2026) reported that supplementation with 12 g/day of dietary fiber for 8 weeks increased HDL levels in both hypertensive and normotensive women. Furthermore, dietary fiber plays an important role in glycemic control. High‐fiber diets have been shown to improve insulin sensitivity and reduce serum glucose levels in diabetic and prediabetic individuals (Mao et al. 2021; Portincasa et al. 2022). The valorization of these by‐products aligns with circular economy principles by converting low‐value residues into functional food ingredients while reducing environmental impacts.

The present study used residues from the processing of mixed fruit juice composed of strawberry, pineapple, and ginger. These fruit residues were selected based on the availability of a mixed processing residue generated by a local juice manufacturer. This approach ensures that the study evaluates a real industrial by‐product, thereby enhancing the practical relevance and applicability of the findings for residue valorization. The valorization of these residues represents a promising approach for reducing environmental impacts and adding value to food‐processing by‐products. Previous studies indicate that a significant proportion of dietary fiber and bioactive compounds (Ghani et al. 2026; Ispiryan and Jarienė 2026), such as phenolic compounds and anthocyanins, remain retained in these residues, highlighting their potential as functional ingredients in the development of new food products (Zamani et al. 2026).

In this context, the production of flour from plant‐based residues through drying and milling enables their incorporation into various food products, such as cookies (Dhillon et al. 2026), cakes (Miri et al. 2025), and meat analogs (Asha et al. 2025). These ingredients can enhance nutritional quality due to their high dietary fiber content and antioxidant compounds, which may positively influence the physicochemical, technological, and sensory properties of foods. The use of residue‐derived flours in plant‐based foods has attracted increasing interest, particularly in products that aim to combine nutritional quality with sensory acceptance (Ghani et al. 2026). Plant‐based burgers stand out among meat analogs due to their growing demand and wide consumer acceptance. Market reports project that the global plant‐based meat sector will grow at an accelerated rate in the coming years, with an estimated annual growth of approximately 19% through 2030, reflecting increasing demand for plant‐based alternatives driven by sustainability, animal welfare, and health‐related motivations (Grand View Research 2024).

Therefore, this study aimed to characterize a flour obtained from mixed fruit juice residues composed of strawberry, pineapple, and ginger and to evaluate its application in the formulation of plant‐based burgers. The effects of mixed fruit juice residue flour incorporation on the physicochemical, technological, nutritional, and sensory properties of the burgers were investigated, thereby contributing to reducing food waste and developing products aligned with current sustainability‐driven demands.

## 2 Material and Methods

### 2.1 Blended Juice Residue

The mixed juice processing residue, composed of strawberry, pineapple, and ginger, was supplied by Super Labs (Eldorado do Sul, RS, Brazil). The material consisted of strawberry residue, peeled pineapple residue, and ginger residue in proportions of 45:45:10% (w/w). This proportion corresponds exactly to the residue blend generated during the commercial juice production process by the supplying company and was therefore used without modification to preserve the industrial relevance of the material. It was stored at −18°C until use.

### 2.2 Blended Residue Flour

The mixed juice residue was subjected to drying in a forced‐air oven at 45°C for 16 h until reaching a final moisture content between 12% and 15%, following Codex recommendations (CODEX ALIMENTARIUS 1985). After drying, the material was milled using a knife mill (Solab, SL‐30, Piracicaba, SP, Brazil) to obtain a powder with particle size <30 mesh. The mixed fruit juice residue flour was vacuum‐packed, protected from light, and stored at room temperature (25°C) until further analyses.

### 2.3 Proximate Composition and Dietary Fiber Analysis of Residue Flour

Moisture (AOAC 925.10), ash (AOAC 923.03), protein (AOAC 920.87), lipid (AOAC 920.85), and pH (AOAC 943.02) were determined according to standard AOAC methods (Association of Official Analytical Chemistry 2005), with carbohydrate content estimated by difference. The dietary fiber content (total, soluble, and insoluble) (AOAC 991.43) was quantified using the enzymatic–gravimetric method (Association of Official Analytical Chemistry 2005). All analyses were performed in triplicate and expressed on a dry matter basis.

#### 2.3.1 Technological Properties of the Mixed Fruit Juice Residue Flour

The mixed fruit juice residue flour was characterized for water‐holding capacity (WHC), oil‐holding capacity (OHC), and water solubility according to previously reported methods (Fernández‐López et al. 2009; Cano‐Chauca et al. 2005). Water solubility was determined from the mass of dissolved solids recovered in the supernatant after centrifugation. All analyses were performed in triplicate.

### 2.4 Evaluation of Antifungal Capacity of the Mixed Fruit Juice Residue Flour

#### 2.4.1 Preparation of the Mixed Fruit Juice Residue Flour Extract

Bioactive compounds were obtained from the mixed fruit juice residue flour using an aqueous extraction method, following the procedure previously described by da Cunha Espinosa et al. (2026). Briefly, mixed fruit juice residue flour samples were dispersed in distilled water (1:10 w/v) and subjected to vortex mixing, ultrasound‐assisted extraction, and centrifugation. The resulting supernatant was filtered through 0.22 µm membranes and stored under refrigeration until further use.

#### 2.4.2 Fungal Strains and Inoculum Preparation

Fungal strains of *Fusarium proliferatum* and *Fusarium verticillioides* were obtained from the Laboratory of Applied Mycology (UFRGS, Porto Alegre, Brazil). Species identification was confirmed by DNA sequencing, and the obtained sequences were compared with those available in the *Fusarium* Multilocus Sequence Typing (MLST) database using the Basic Local Alignment Search Tool (BLAST), as described by Machado et al. (2022). For inoculum preparation, strains were cultured on potato dextrose agar (PDA) under controlled conditions for 72 h to obtain actively growing mycelia.

#### 2.4.3 Mycelial Growth Inhibition Assay

Antifungal activity was evaluated using a mycelial growth inhibition assay on PDA, based on previously reported procedures (Rizzello et al. 2017). The mixed fruit juice residue flour extract was incorporated into the culture medium at different concentrations (2.5% and 5%), and fungal growth was monitored during incubation. Colony development was assessed by measuring diameters, and all assays were performed in triplicate. The inhibition percentage (IP, %) was calculated using the following equation:

$$\mathrm{IP}\;\;\left(\%\right)=\frac{\left(C-T\right)}{C}\;\times 100$$

where *C* is the average colony diameter (mm) in the control, and *T* is the average colony diameter (mm) in the treatment.

### 2.5 Bioactive Compounds of the Mixed Fruit Juice Residue Flour

#### 2.5.1 Identification and Quantification of Phenolic Compounds

Phenolic compounds were extracted using hydroalcoholic solution (80% methanol acidified with formic acid 0.35%) under exhaustive extraction conditions, and the samples were filtered through a cellulose acetate membrane (0.22 µm). Identification and quantification were performed by a high‐performance liquid chromatography (HPLC) system (Waters Corporation, Alliance 2695, Milford, MA, USA) using a C18 column (250 mm × 4.6 mm, 4 µm; Waters Corporation, Milford, MA, USA) and diode array detection (Mallmann et al. 2020).

Chromatographic separation was carried out using a binary gradient system with a mobile phase system consisting of solvent A (acidified water with 0.1% formic acid) and solvent B (acetonitrile with 0.1% formic acid). The elution was performed starting at 5% B and increasing to 50.2% B over 46 min, with the flow rate of 0.5 mL min^−^^1^ and an injection volume of 20 µL. The chromatograms were monitored at 280 nm, and the quantification was performed using external calibration curves of pyrogallic acid, dimethoxy‐hydroxycinnamic acid, and chlorogenic acid, as described in Table 1. All analyses were performed in triplicate (*n* = 3), and all solvents were of HPLC grade.

**TABLE 1:** Analytical calibration parameters for phenolic compounds and anthocyanidin aglycones, including concentration range, coefficient of determination (*R* ^2^), limit of detection (LOD), and limit of quantification (LOQ).

	Concentration range (mg/L)	*R* ^2^	LOD (mg/L)		LOQ (mg/L)
Pyrogallic acid	14–140	0.9983	5.59	16.95	
Dimethoxyhydroxycinnamic acid	11.1–111	0.9991	6.25	18.94	
Chlorogenic acid	11.6–116	0.9993	3.52	10.68	
Pelargonidin aglycone	6.07–72.88	0.9980	3.95	11.98	
Cyanidin aglycone	4.81–19.25	0.9906	2.88	8.72	

#### 2.5.2 Identification and Quantification of Anthocyanins

Anthocyanins were extracted from mixed fruit juice residue flour using methanol containing 1% (v/v) HCl and homogenization with an Ultra‐Turrax homogenizer (IKA, T25, Staufen, Germany). The extracts were concentrated using a rotary evaporator. Anthocyanins were quantified by HPLC and identified by comparison with external standards.

HPLC analysis was performed using an HPLC system (Agilent Technologies, Agilent 1100 Series, Santa Clara, CA, USA) equipped with a quaternary pump and a UV–Vis detector. Pigments were separated on a reversed‐phase C18 column (CLC‐ODS, 5 µm, 250 × 4.6 mm; Shimadzu, Kyoto, Japan). The mobile phase consisted of water acidified with 5% formic acid (A) and methanol (B). Elution was carried out using a linear gradient from 85:15 to 20:80 (A: B, v/v) over 20 min. The flow rate was 1.0 mL min^−^^1^, and the injection volume was 5 µL. The column temperature was maintained at 29°C, and detection was performed at 520 nm.

All analyses were performed in triplicate. Anthocyanins were identified by comparing sample retention times with those of commercial standards (Sigma‐Aldrich, St. Louis, MO, USA). Quantification was performed using external calibration curves constructed with anthocyanin standards, including pelargonidin aglycone, cyanidin aglycone, delphinidin aglycone, pelargonidin 3,5‐diglycoside, and pelargonidin‐3‐glucoside (Table 1).

### 2.6 Oxygen Radical Absorbance Capacity (ORAC) of Mixed Fruit Juice Residue Flour

The peroxyl radical (ROO•) scavenging capacity was determined by monitoring the fluorescence decay resulting from ROO•‐induced degradation of fluorescein in the presence of sample extracts. Quantification was performed according to Vargas et al. (2024) with modifications. In each well of a black microplate, 25 µL of sample, blank, or standard was combined with 150 µL of fluorescein solution and incubated at 37°C for 10 min. After adding 25 µL of AAPH solution, fluorescence decay was measured for 90 min at 37°C using a microplate reader (PerkinElmer, Enspire 2300, Waltham, MA, USA). Excitation and emission wavelengths were set to 530 and 485 nm, respectively. A Trolox calibration curve was prepared using concentrations ranging from 2 to 36 µmol L^−^^1^. Results were expressed as µmol Trolox equivalents per gram of sample (µmol TE.g^−^^1^).

### 2.7 Development and Technological Application in Plant‐Based Burgers

#### 2.7.1 Formulation and Preparation of Plant‐Based Burgers

To develop plant‐based burgers containing flour derived from strawberry, pineapple, and ginger, ingredients were purchased from a local market in Porto Alegre, RS, Brazil, and the mixed fruit juice residue flour was prepared at the Bioactive Compounds Laboratory of the Federal University of Rio Grande do Sul (UFRGS). The formulations of the control burger and the two formulations containing mixed fruit juice residue flour were prepared following the formulations of Table 2.

**TABLE 2:** Formulation of the control burger (0%) and plant‐based burgers containing 10% and 15% replacement of flours with mixed fruit juice residue flour.

Ingredients (%)	H0	H10	H15
Cooked chickpeas	100	100	100
Residue flour	0	10	15
Oatmeal (Nestlé)	5	0	0
Brown flaxseed flour (Geração Saúde)	5	0	0
Buckwheat flour (Ecobio)	8.75	8.75	3.75
Onion	25	25	25
Garlic	0.8	0.8	0.8
Salt (Cisne)	1.4	1.4	1.4
Lemon pepper (Bombay)	0.8	0.8	0.8
Chimichurri (Terra Rica)	0.8	0.8	0.8
Liquid smoke (Gonzalo)	0.8	0.8	0.8
Beet flour (Soldiers)	0.2	0.2	0.2
Water	20	20	20

Abbreviations: H0, control plant‐based burger without flour addition; H10, burger with 10% mixed fruit juice residue flour substitution; H15, burger with 15% mixed fruit juice residue flour substitution.

It should be noted that the control formulation contained oatmeal and brown flaxseed flour, which were completely replaced by mixed fruit juice residue flour in the H10 and H15 formulations. Therefore, the results should be interpreted as comparisons among different formulations rather than as representing the isolated effect of mixed fruit juice residue‐flour incorporation. This formulation strategy was adopted to maintain a constant total flour content across all formulations while evaluating the technological feasibility of using mixed fruit juice residue flour as a complete substitute for the original flour blend.

#### 2.7.2 Proximate Composition of Plant‐Based Burgers

Burgers containing mixed fruit juice residue flour were analyzed for pH (AOAC 943.02), moisture (AOAC 925.10), and ash (AOAC 923.03) according to AOAC (Association of Official Analytical Chemistry 2005). All these analyses were performed in triplicate. Lipid, fiber, and protein contents were calculated based on the nutritional composition of the ingredients used, and carbohydrate content was determined by difference on a dry matter basis.

#### 2.7.3 Physical Analysis of Plant‐Based Burgers

Sample diameter and height were measured after cooking using a digital caliper, taking three measurements at different points for each sample. Sample weight was determined before and after cooking using an analytical balance.

#### 2.7.4 Textural Profile Analysis

After cooking, burgers were analyzed by texture profile analysis to determine their textural properties. A texture analyzer (TA.TX plus; Stable Micro Systems Ltd., Surrey, United Kingdom) was used, with samples approximately 3 cm in diameter and 1 cm in height (*n* = 5). The test consisted of two compression cycles to 50% deformation using a cylindrical probe (3.6 cm diameter) at a crosshead speed of 2 mm s^−1^. The parameters of hardness, springiness, cohesiveness, gumminess, and chewiness were calculated according to Bourne (1990).

#### 2.7.5 Sensory Analysis

A sensory evaluation was conducted to determine the acceptance of burgers formulated with mixed fruit juice residue flour. The test involved 97 untrained consumers, who assessed the samples based on appearance, color, aroma, flavor, aftertaste, texture, and overall liking. Both vegetarian and non‐vegetarian consumers were included in the study, as no dietary restrictions were established for participation. A nine‐point hedonic scale was applied, with the following anchors: 1 = disliked extremely, 2 = disliked very much, 3 = disliked moderately, 4 = disliked slightly, 5 = neither liked nor disliked, 6 = liked slightly, 7 = liked moderately, 8 = liked very much, and 9 = liked extremely, in accordance with Dutcosky (2015). Participants also indicated their purchase intention through a direct question regarding their willingness to buy the product. The results were expressed as percentages and calculated using the acceptance index (AI), as shown below:

$$\mathrm{Acceptance}\;\mathrm{index}\;\left(\mathrm{AI}\right)=\frac{M\;\times 100}{9}$$

where *M* corresponds to the mean score obtained for each attribute, and 9 represents the maximum value of the hedonic scale.

The sensory evaluation was conducted only with the formulations containing mixed fruit juice residue flour (10% and 15%), since the objective was to compare consumer acceptance between the proposed inclusion levels and identify the most suitable concentration of the ingredient in the developed plant‐based burgers.

#### 2.7.6 Sensory Panel and Ethical Approval

The sensory evaluation was conducted with 97 untrained panelists recruited from the Federal University of Rio Grande do Sul. All participants were informed about the objectives and procedures of the study and provided written informed consent prior to participation.

The study was conducted in accordance with the ethical principles for research involving human subjects and was approved by the Ethics Committee of the Federal University of Rio Grande do Sul (*Comitê de Ética em Pesquisa—CEP/UFRGS*) under approval number 36 691 414.1.0000.5347.

Samples were coded with three‐digit random numbers and presented to panelists in a randomized order. The evaluations were conducted in individual booths under white light at room temperature (22 ± 2°C). Filtered water was provided for palate cleansing between samples. The questionnaires were administered using paper forms, and data were collected directly for subsequent analysis.

Participants were instructed on the evaluation procedures and assessed the samples under controlled conditions using a nine‐point hedonic scale for attributes including appearance, aroma, texture, flavor, and overall acceptability.

### 2.8 Statistical Analysis

Data were analyzed by analysis of variance (ANOVA), followed by Tukey's test at a 5% significance level, using Statistica 13.0 software (StatSoft, Inc.).

## 3 Results and Discussion

### 3.1 Proximate Composition and Dietary Fiber Analysis of Mixed Fruit Juice Residue Flour

**FIGURE 1:**
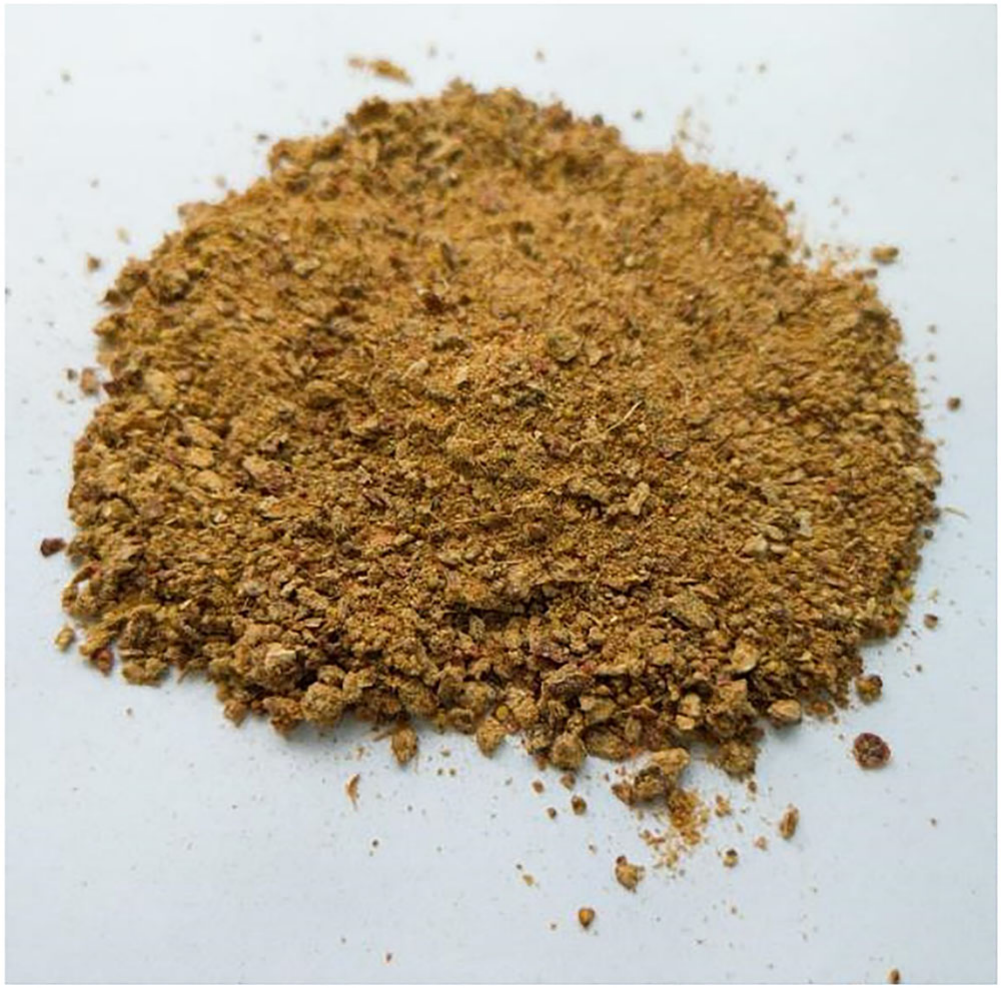
Appearance of flour produced from pineapple, strawberry, and ginger juice processing residues.

The appearance of the mixed fruit juice residue flour obtained from pineapple, strawberry, and ginger juice processing residues is shown in Figure 1. The pH was 2.87 ± 0.53, reflecting the natural acidity of the fruit residues used in its production. The proximate composition is presented in Table 3. The moisture content was 12.17 g/100 g DM, similar to those values reported for other fruit residues, such as pineapple (13.56 g/100 g DM) and papaya peel (15.85 g/100 g DM) (Crizel et al. 2016). The lipid content was low (3.21 g/100 g), as expected, since the mixed fruit juice residue flour was produced exclusively from fruit pomace (strawberry and pineapple). These values are consistent with those reported for flours from other fruit by‐products, such as mango (2.2 g/100 g DM), apple (4.46 g/100 g DM), and orange (1.83 g/100 g DM) (Ajila et al. 2008; Crizel et al. 2013; Figuerola et al. 2005). Lipid content in such residues varies depending on the extraction processes used in the fruit industry and the type of by‐product (peel, seed, or pit).

**TABLE 3:** Proximate composition and functional properties of the mixed fruit juice residue flour (dry matter).

Proximate composition (g/100 g)	
Moisture (%)	12.17 ± 0.19
Ash (%)	5.45 ± 0.11
Lipids (%)	3.21 ± 0.10
Protein (%)	2.34 ± 0.42
Soluble dietary fiber (%)	8.44 ± 0.41
Insoluble dietary fiber (%)	27.32 ± 1.80
Total dietary fiber (%)	35.76 ± 1.40
Carbohydrates^a^	52.48 ± 0.22

Functional properties	
Water solubility (%)	44.99 ± 0.48
WHC (g water g^−1^ flour)	5.12 ± 0.54
OHC (g oil g^−1^ flour)	3.53 ± 0.08

^a^Calculated by difference.

Abbreviations: WHC, water‐holding capacity; OHC, oil‐holding capacity.

Protein content varies depending on the flour source, and fruit residues generally present low levels of this nutrient. The protein content of the mixed fruit juice residue flour obtained in this study was 2.34 g/100 g DM, which is lower than that reported for orange (6.56 g/100 g DM), lime (6.75 g/100 g DM), and peach (7.38 g/100 g DM) residues (Durán‐Aranguren et al. 2023).

Fruit and vegetable by‐products are recognized as rich sources of dietary fiber. The flour obtained from pineapple, strawberry, and ginger juice processing residues showed a high total dietary fiber content (35.76 g/100 g DM) (Table 3). The mixed fruit juice residue flour presented high dietary fiber content, consistent with previous findings reported by da Cunha Espinosa et al. (2026), although differences may be attributed to variations in raw material composition. This value is lower than those reported by Dalal et al. (2020) for residues from other fruits, such as banana (50%), citrus peels (54.82%), peach (54.5%), and mango peel (51.2%). Dietary fiber content in by‐products may vary depending on fruit variety and the type of residue, as composition differs among peel, pomace, and seeds. The insoluble fiber fraction predominated, accounting for approximately 76% of the total dietary fiber. The high insoluble dietary fiber (IDF) content indicates the presence of substantial amounts of cellulose and hemicellulose (Martínez et al. 2012). The soluble fiber content (8.44 g/100 g DM) was higher than that reported for other fruit pomace residues, such as blueberry (1.70 g/100 g DM) and raspberry (2.12 g/100 g DM) (Šarić et al. 2019).

Soluble dietary fiber exhibits several technological properties, including gelling, water‐binding, thickening, and emulsifying capacity, enabling its use as a fat replacer and foam stabilizer. In contrast, insoluble fiber is primarily associated with structural functions, contributing to stabilization, texturizing, moisture control, reducing shrinkage, delaying staling, and improving product stability (Santos et al. 2022). Functional properties of dietary fiber are influenced by the IDF/SDF ratio, particle size, extraction conditions, polysaccharide structure, and botanical source. Properties such as water and oil‐holding capacities and solubility are particularly important, as they determine its functionality in food applications.

WHC is the amount of water retained by hydrated fiber under external force, such as pressure or centrifugation. The mixed fruit juice residue flour showed a WHC of 5.12 g water/g flour, a value comparable to that reported by Viuda‐Martos et al. (2012) for pomegranate juice (4.9 g water/g dry fiber). Lower WHC values have been reported for raspberry and blueberry pomace (2.10 and 3.07 g/g, respectively) (Šarić et al. 2019), which may be associated with differences in fiber composition and structural characteristics of the fruit matrix, as well as processing conditions affecting water‐binding properties (Rabetafika et al. 2014). High WHC is associated with improved technological properties, such as reduced syneresis and enhanced viscosity and texture of food products (Zayas 1997).

OHC depends on the chemical and physical structure of polysaccharides and plays an important role in fat retention during processing, contributing to flavor preservation. The mixed fruit juice residue flour showed an OHC of 3.53 g oil/g flour, higher than values reported for apple pomace (1.3–1.6 g/g) (Gorjanović et al. 2020). This behavior may be associated with the high IDF content, as IDF can retain several times its mass in oil. Additionally, particle size may influence this property, with smaller particles exhibiting higher OHC due to increased surface area (Viuda‐Martos et al. 2012). The solubility of the mixed fruit juice residue flour was 44.99% (Table 3), exceeding values reported for flours derived from other fruit residues, such as orange (28.95%), blueberry (40.15%), and olive (24.19%) (Crizel et al. 2016).

### 3.2 Antifungal Capacity of the Mixed Fruit Juice Residue Flour

**FIGURE 2:**
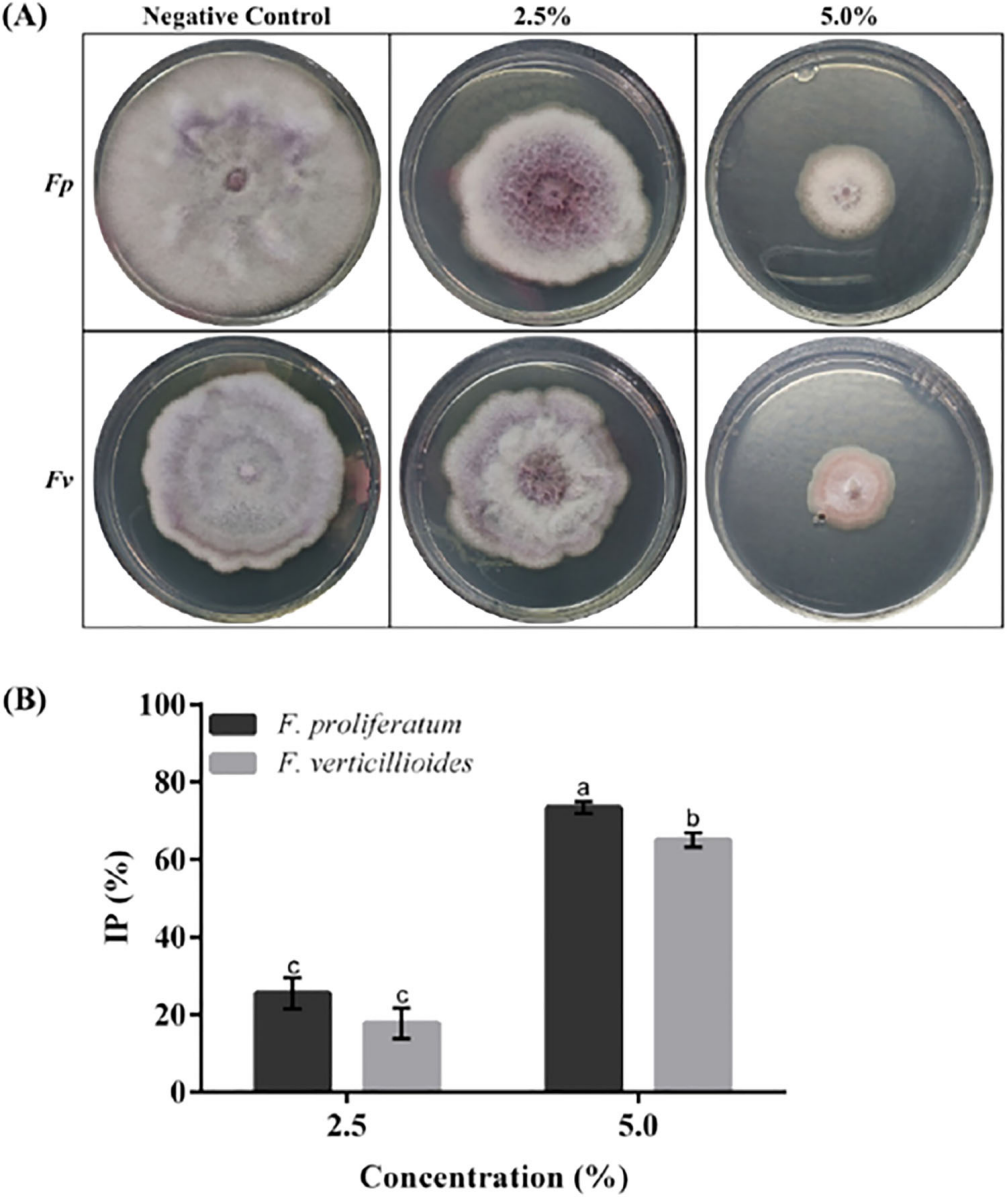
Antifungal activity of mixed fruit juice residue flour extract against *Fusarium proliferatum* (Fp) and *Fusarium verticillioides* (Fv). (A) Representative images of mycelial growth on PDA plates under different treatments. (B) Mycelial growth inhibition (IP, %) of *F. proliferatum* and *F. verticillioides* at 2.5% and 5.0% extract concentrations. Results are expressed as mean ± standard deviation (*n* = 3). Different lowercase letters indicate significant differences among treatments according to Tukey's test (*p* < 0.05).

Results showed a concentration‐dependent inhibition of mycelial growth (Figure 2). At 2.5%, inhibition reached 25.53 for *F. proliferatum* and 17.77 for *F. verticillioides*, with no statistically significant difference between species (*p* > 0.05). At 5.0%, inhibition increased significantly (*p* < 0.05) to 73.38 and 65.11, respectively. Two‐way ANOVA indicated that both concentration and fungal species significantly affected mycelial inhibition, with no significant interaction effect. These findings suggest that the extract exerts a consistent antifungal effect across species, with concentration as the primary determinant of efficacy.

The increase in antifungal activity observed at 5.0% can be attributed to the phenolic compounds present in the mixed fruit juice residue flour. The extract contained phenolic acids and flavonoids, including p‐coumaric acid, chlorogenic acid, protocatechuic acid, catechin, and epicatechin, which are recognized contributors to antifungal activity. In pineapple matrices, hydroxycinnamic acids such as p‐coumaric acid have demonstrated strong inhibitory effects against *Fusarium* spp., including complete inhibition of mycelial growth at higher concentrations (Barral et al. 2017). Similarly, chlorogenic acid has been widely associated with antifungal activity, acting through mechanisms such as membrane permeabilization and cell lysis, thereby impairing fungal development (Martínez et al. 2017). Additionally, chlorogenic and caffeic acid derivatives can inhibit fungal growth and mycotoxin production, underscoring their role as natural defense compounds against *Fusarium* spp. (Gauthier et al. 2016; Kulik et al. 2017). Flavonoids, including catechin and epicatechin, have also been linked to increased resistance to *Fusarium* infection, as their accumulation correlates with reduced fungal abundance and disease incidence (Kong et al. 2025). Overall, the antifungal activity observed in this study appears to result from the combined effects of these phenolic compounds.

The antifungal activity of the mixed fruit juice residue flour is particularly relevant for its incorporation into plant‐based burger formulations. Agro‐industrial by‐products rich in phenolic compounds have been successfully applied in meat and meat analog systems, contributing to microbial control and product stability without compromising sensory attributes (Gonçalves et al. 2021). Likewise, extracts from fruit residues, including passion fruit by‐products, have demonstrated the ability to inhibit microbial growth and improve the microbiological stability of burger systems during storage (de Souza et al. 2022). In this context, the observed antifungal effect underscores the potential of the mixed fruit juice residue flour as a functional and natural preservative in plant‐based foods, contributing to shelf‐life extension and enhanced microbiological safety, in line with clean label trends.

### 3.3 Identification and Quantification of Phenolic Compounds in the Mixed Fruit Juice Residue Flour

Seven phenolic compounds were identified in the mixed fruit juice residue flour (Table 4), with protocatechuic acid, p‐coumaric acid, and catechin being the most abundant. Da Cunha Espinosa et al. (2026) reported a broader phenolic profile comprising ten compounds, which may be attributed to differences in raw material composition and processing conditions. Comparable phenolic profiles have been reported in fruit by‐products, such as mango seed residues (Alañón et al. 2021), where p‐coumaric acid was detected at 4.36 mg/100 g DM (at the ripening stage with the highest concentration and in only one cultivar), and catechin was found in all varieties and ripening stages, ranging from 3.28 to 50.23 mg/100 g DM. The presence of these compounds in the studied flour indicates that phenolic constituents were preserved despite the thermal drying process, highlighting its potential as a source of bioactive compounds.

**TABLE 4:** Identification and quantification of phenolic compounds and anthocyanins of mixed fruit juice residue flour by HPLC.

Retention time (min)	Phenolic compounds	Mixed fruit juice residue flour (mg/100 g DM)
18.07	3,5‐Dihydroxybenzoic acid	17.70 ± 2.02
30.81	p‐Coumaric acid	170.15 ± 10.35
32.25	3,5‐dimethoxy‐4‐hydroxycinnamic acid	0.74 ± 0.11
22.35	Catechin	37.39 ± 8.55
25.02	Epicatechin	30.91 ± 3.73
21.92	Chlorogenic acid	21.80 ± 1.09
18.84	Protocatechuic acid	234.55 ± 8.07
	**Total**	**513.24 ± 16.27**

**Retention time (min)**	**Anthocyanins**	**Mixed fruit juice residue flour** **(mg/100 g DM)**
7.5	Cyanidin aglycone	3.16 ± 0.34
8.8	Pelargonidin aglycone	3.99 ± 0.76
	**Total**	**7.15 ± 1.1**

Similar results were reported by Pukalskienė et al. (2021), who analyzed strawberry pomace from the juice industry, dried at 40°C for 48 h, and extracted using different methods. Catechin and p‐coumaric acid were identified as common compounds, although at relatively low concentrations (0.1 and 1.90 mg/100 g DM, respectively, in solid–liquid extraction with water). These lower values may be partially explained by the presence of bound phenolics, as compounds such as p‐coumaric and ferulic acids are often esterified to cell wall polysaccharides, limiting their extraction under mild conditions. In line with this, Campos et al. (2020) identified chlorogenic acid in pineapple residues, which was also detected in the present study. Overall, these findings support the hypothesis that blending different by‐products contributes to a broader and more diverse phenolic profile.

### 3.4 Identification and Quantification of Anthocyanins in the Mixed Fruit Juice Residue Flour

Table 4 summarizes the anthocyanin composition of the flour obtained from pineapple, strawberry, and ginger juice processing residues. The total anthocyanin content was 7.15 mg/100 g DM, with only two compounds identified and quantified: pelargonidin aglycone (3.99 mg/100 g DM) and cyanidin aglycone (3.16 mg/100 g DM).

The presence of these compounds is consistent with the contribution of strawberry, a known source of pelargonidin. In fruits, anthocyanins are typically present in glycosylated forms, which enhance their stability and solubility. However, during processing, these compounds may undergo cleavage, leading to the formation of aglycone forms (Wallace and Giusti 2015), which may explain their detection in the mixed fruit juice residue flour. Thus, the anthocyanin profile observed here likely reflects not only the original composition of the raw materials but also transformations occurring during processing. Nevertheless, their occurrence remains relevant, particularly due to their role in color and their association with antioxidant properties.

Results reported in the literature show considerable variability in anthocyanin composition. Vargas et al. (2016) identified pelargonidin‐3‐glucoside as the sole anthocyanin in strawberry processing residues at a concentration of 71.86 mg/100 g DM. Such differences are commonly attributed to factors such as cultivar, growing conditions, and extraction procedures (Moyer et al. 2002). Additionally, delphinidin (aglycone), pelargonidin‐3‐glucoside, and pelargonidin‐3,5‐diglucoside were detected in the mixed fruit juice residue flour; however, their concentrations were below the limits of quantification.

Processing may further influence this profile by affecting compound stability and promoting the degradation of bioactive constituents. Accordingly, the anthocyanin profile observed in the mixed fruit juice residue flour should be interpreted considering both compositional and processing‐related factors. Bioactive compounds in fruits and vegetables, particularly phenolics and anthocyanins, play an important role in antioxidant activity through free radical scavenging and the mitigation of oxidative stress (Dhalaria et al. 2020).

### 3.5 Oxygen Radical Absorbance Capacity (ORAC) of the Mixed Fruit Juice Residue Flour

The antioxidant capacity of the flour obtained from mixed juice residues (strawberry, pineapple, and ginger) was evaluated using the ORAC assay, which assesses the ability of compounds to neutralize peroxyl radicals via hydrogen atom transfer mechanisms (Rangani and Ranaweera 2023). The sample exhibited an ORAC value of 377.21 µmol TE g^−^^1^, indicating a high antioxidant potential. This finding reflects the ability of the extract to delay fluorescein oxidation over time (Hadidi et al. 2022). Such activity can be attributed to its composition, as these residues are rich in phenolic compounds, including anthocyanins and flavonoids, which are well known for their antioxidant properties (Da Silva et al. 2025; Nogueira et al. 2025).

Compounds derived from ginger exhibit protective effects against oxidative stress, which is linked to the overproduction of reactive oxygen species. In a comparative study, freeze‐dried ginger extract showed an antioxidant capacity of 227.79 ± 10.40 µmol TE g^−^^1^, whereas fresh rhizome exhibited higher values (414.37 µmol TE g^−^^1^) (Dalsasso et al. 2022; Russo et al. 2023). Similarly, Nemzer et al. (2018) investigated strawberry drying processes and reported an antioxidant capacity of 412.50 µmol TE g^−^^1^ in extracts obtained by hot air drying (70°C for 16 h). In another study, Da Silva et al. (2025) applied 1% strawberry extract to pork burgers and observed higher antioxidant activity compared to jabuticaba extract (15.64 µmol TE mL^−^^1^).

Pereira et al. (2024) evaluated hot air‐dried pineapple flour (60°C for 48 h) and reported an antioxidant capacity of 20.45 µmol TE g^−^^1^. Agro‐industrial residues often contain high levels of phenolic compounds bound to the dietary fiber matrix, which may limit their immediate extractability (Li et al. 2017). However, processing steps such as drying and milling during mixed fruit juice residue flour production can disrupt the plant cell wall structure, reduce particle size, and promote the release of bound phenolics, thereby enhancing their bioaccessibility (Li et al. 2016). In this context, Tamkutė et al. (2022) incorporated cranberry residue extract into hamburgers and observed a fourfold increase in antioxidant activity, from 13.10 to 54.26 µmol TE mL^−^^1^. These findings further support the potential of agro‐industrial residues as sources of bioactive compounds for application in functional foods.

### 3.6 Development and Technological Application in Plant‐Based Burgers

To explore the application of mixed fruit juice residue flour as a value‐added ingredient, chickpea‐based burgers were developed, given the increasing demand among vegan and vegetarian consumers. Two formulations were prepared using an ingredient substitution approach to maintain characteristics similar to the control formulation.

**FIGURE 3:**
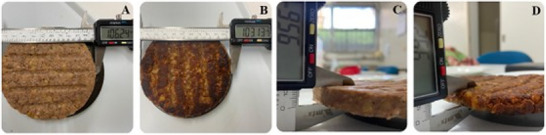
Visual appearance of raw (A and C) and cooked (B and D) plant‐based burgers.

Based on preliminary visual inspection by the research team, both formulations resulted in visually suitable products (Figure 3), compared to the control and commercial burgers. Flavor and aroma were mainly influenced by the ginger in the mixed fruit juice residue flour, as reported by panelists in the open comments of the sensory evaluation form. The presence of residual flavors from the incorporated mixed fruit juice residue flour may represent a potential risk for consumer acceptance.

### 3.7 Proximate Composition of Plant‐Based Burgers

Table 5 presents the results of the analyses performed on the burgers. The moisture content was consistent with values reported for similar products. Salvadori et al. (2025) evaluated plant‐based burgers formulated with pine nuts and green banana biomass and reported moisture contents ranging from 51.09% to 63.15%, comparable to commercial beef and vegan burgers. Similarly, de Oliveira et al. (2026) analyzed soy‐based burgers incorporating extracts from non‐conventional plants and reported moisture contents between 53% and 60%. These findings agree with the results of the present study.

**TABLE 5:** Proximate composition of plant‐based burger with the mixed fruit juice residue flour substitution (g per 100 g).

Parameter	H0	H10	H15
Moisture (%)	61.04 ± 0.64	61.32 ± 0.63	62.33 ± 0.97
Ash	1.70 ± 0.15	2.95 ± 0.04	3.36 ± 0.02
Lipids	3.40	2.09	2.10
Protein	8.21	7.21	6.85
Total dietary fiber	7.19	8.28	8.99
Carbohydrates	79.48	79.47	78.69
Caloric value (kcal/100 g)	395.82	382.11	379.08

Abbreviations: H0, control plant‐based burger without mixed fruit juice residue flour addition; H10, burger with 10% mixed fruit juice residue flour substitution; H15, burger with 15% mixed fruit juice residue flour substitution. Moisture and ash were analyzed in triplicate; lipids, protein, dietary fiber, carbohydrates, and caloric value were estimated from food composition tables (Section 2.7.2). Values represent experimental and calculated data with different statistical properties; therefore, inferential statistical analysis was not applied.

Ash content increased with the level of incorporation of the mixed fruit juice residue flour, indicating a greater mineral contribution in formulations with higher flour addition. The values obtained are comparable to those reported by Kea et al. (2025) for chickpea burgers with textured vegetable protein, which ranged from 2.92 to 3.74 g/100 g depending on the formulation. Similarly, Fernandes et al. (2024) observed an increase in ash content from 3.42 to 5.12 g/100 g in plant‐based burgers containing Baru nut residue compared to those formulated with textured pea protein. These findings suggest that incorporating agro‐industrial residues can enhance the mineral content and nutritional value of plant‐based products. A similar trend was observed for pH, which decreased with increasing levels of mixed fruit juice residue flour, with values of 5.49 (H0), 4.93 (H10), and 4.60 (H15), all of which were significantly different from each other. This behavior is consistent with the acidic nature of the mixed fruit juice residue flour (pH 2.87), derived from fruit residues.

The substitution of conventional flours with mixed fruit juice residue flour affected the nutritional composition of the burgers. A reduction of approximately 40% in total lipid content was observed, mainly due to replacing brown flaxseed flour, which is rich in lipids (38.6 g/100 g, according to manufacturer data). In contrast, total dietary fiber content increased by approximately 15% (H10) and 25% (H15) compared to the control formulation. These values are comparable to those reported by Biazotto et al. (2025) for commercial plant‐based burgers, which ranged from 6.88 to 10.62 g/100 g depending on composition. This similarity suggests that both the plant matrix and the incorporation of fiber‐rich ingredients, such as agro‐industrial residues, play a key role in determining the final fiber content. Consequently, the addition of mixed fruit juice residue flour improved the nutritional profile of the burgers, aligning them with commercial products.

No significant differences in carbohydrate content were observed among the formulations. A slight reduction in caloric value was observed, with decreases of approximately 13 kcal/100 g (H10) and 16 kcal/100 g (H15) compared to the control, with minimal difference between the substituted formulations. Protein content decreased as the level of mixed fruit juice residue flour substitution increased, with reductions of approximately 12% (H10) and 16% (H15) compared to the control. However, these values are consistent with plant‐based burgers formulated without isolated proteins. Salvadori et al. (2025) reported protein contents ranging from 6.64 to 8.19 g/100 g in burgers based on pine nuts and green banana biomass. Higher protein levels are typically observed when isolated plant proteins are incorporated. For example, Kea et al. (2025) reported values ranging from 13.22 to 17.11 g/100 g in chickpea‐based burgers supplemented with textured vegetable protein. These findings indicate that the lower protein content observed in the present study is primarily related to the formulation strategy rather than the incorporation of residue flour itself.

### 3.8 Physical Analysis of Plant‐Based Burgers

The physical analyses conducted before and after cooking indicated similar behavior across all three burger formulations. Cooking weight loss was 5.68% ± 0.24 for the control formulation, compared to formulations containing 10% and 15% mixed fruit juice residue flour (2.13% ± 1.87 and 2.37% ± 1.10, respectively). This reduction in water loss can be attributed to the high fiber content and WHC of the mixed fruit juice residue flour, which helps retain moisture and maintain the structural integrity of the product during cooking (De Marchi et al. 2021).

Regarding dimensional changes, no significant differences (*p* ≥ 0.05) were observed among the formulations for diameter variation (+0.80% ± 0.78, +2.23% ± 0.84, and +0.67% ± 2.47 for H0, H10, and H15, respectively). The small variations observed may result from both the intrinsic characteristics of the product matrix and handling during grilling, such as turning the burgers with utensils such as spatulas, which can lead to minor deformation of the structure. Overall, the incorporation of up to 15% mixed fruit juice residue flour did not compromise physical stability, preserving dimensional consistency and technological yield during cooking.

### 3.9 Textural Profile Analysis

The addition of flour derived from mixed juice residues influenced the textural profile of plant‐based burgers, particularly hardness, which decreased progressively with increasing incorporation levels, reaching the lowest value in the 15% residue formulation (Table 6). This trend suggests that the fiber‐rich fraction disrupted the matrix's structural organization, likely reducing the network's continuity and, in turn, its resistance to compression. In meat analogues, texture is closely linked to the formation of a protein network capable of withstanding mechanical stress, and alterations in this network result in measurable changes in firmness (Nasrollahzadeh et al. 2024).

**TABLE 6:** Results of the textural analysis of a plant‐based burger with mixed fruit juice residue flour substitution.

Textural parameters	Cohesiveness	Springiness (mm)	Hardness (N)	Gumminess (N)	Chewiness (J)
H0	0.79 ± 0.18^a^	1.19 ± 0.02^a^	58.54 ± 4.80^a^	46.84 ± 13.28^a^	0.056 ± 0.02^a^
H10	0.73 ± 0.34^a^	1.17 ± 0.08^a^	52.57 ± 2.13^b^	39.00 ± 18.06^a^	0.047 ± 0.02^a^
H15	0.97 ± 0.14^a^	1.21 ± 0.01^a^	42.16 ± 2.40^c^	41.26 ± 6.06^a^	0.050 ± 0.01^a^

Abbreviations: H0, control plant‐based burger without mixed fruit juice residue flour addition; H10, burger with 10% mixed fruit juice residue flour substitution; H15, burger with 15% mixed fruit juice residue flour substitution. Different letters within the same column indicate significant differences according to Tukey's test (*p* < 0.05).

In plant‐based matrices, fibers and polysaccharides integrate into the microstructure as dispersed phases, rather than acting as inert fillers. Their incorporation can limit protein–protein interactions and redistribute stress within the three‐dimensional network, promoting less dense structures while maintaining overall cohesion. Such structural reorganization has been widely associated with protein–polysaccharide interactions in structured foods, particularly in plant‐based systems (Loveday 2019; Sha and Xiong 2020).

In addition to structural modifications, water retention plays a key role in the observed mechanical response. Dietary fibers with high hydration capacity alter the balance between solid and aqueous phases, increasing the amount of immobilized water within the matrix. This retained water acts as a structural modulator, reducing resistance to deformation and contributing to less rigid structures after thermal processing, as reported in studies on structured plant proteins and meat analogs (Fiorentini et al. 2020; McClements 2020).

Despite the reduction in hardness, gumminess, and chewiness remained statistically stable across formulations, indicating that the energy required for product breakdown during mastication was preserved. This suggests that structural modifications primarily affected initial resistance to compression without substantially altering mechanical behavior during consumption. In plant‐based products, the preservation of these parameters reflects adequate technological performance since texture perception depends on the structural response throughout mastication (Kyriakopoulou et al. 2021; Sha and Xiong 2020). Similarly, springiness and cohesiveness remained consistent among formulations, showing that the incorporation of mixed fruit juice residue flour did not compromise matrix integrity. Fibrous particles appear to promote internal reorganization without causing collapse of the structural network, consistent with previous observations in structured plant‐based matrices containing particulate phases (Dekkers et al. 2018).

Overall, the combined textural parameters indicate that incorporation of mixed fruit juice residue flour induces a controlled structural reorganization, characterized by reduced initial rigidity while maintaining mechanical cohesion. This balance between softness and structural stability supports the technological feasibility of incorporating up to 15% residue, producing characteristics compatible with commercially available plant‐based burgers.

### 3.10 Sensory Analysis

The results of the sensory analysis are presented in Table 7, showing the mean scores for the evaluated attributes and their respective acceptance percentages. Five of the seven attributes analyzed achieved acceptance above 80%, indicating excellent overall sensory acceptance regardless of the level of incorporated mixed fruit juice residue flour. The attributes of texture and aftertaste, however, showed slightly lower acceptance, with mean scores of 6.72 for the burger containing 10% mixed fruit juice residue flour and 6.56 for the formulation with 15% addition. No significant differences (*p* ≥ 0.05) were observed between the H10 and H15 formulations for texture and aftertaste scores.

**TABLE 7:** Results of the sensory analysis of plant‐based burger with mixed fruit juice residue flour substitution.

Attributes	H10		H15	
	Assigned score (M ± SD)	Acceptance index (%)	Assigned score (M ± SD)	Acceptance index (%)
Appearance	7.36 ± 1.50^a^	81.79^a^	7.32 ± 1.59^a^	81.33^a^
Color	7.62 ± 1.43^a^	84.65^a^	7.58 ± 1.36^a^	84.19^a^
Aroma	7.91 ± 1.31^a^	87.86^a^	7.67 ± 1.36^a^	85.22^a^
Flavor	7.70 ± 1.37^a^	85.57^a^	7.23 ± 1.58^b^	80.30^b^
Aftertaste	7.16 ± 1.59^a^	79.61^a^	6.92 ± 1.78^a^	76.86^a^
Texture	6.72 ± 1.87^a^	74.68^a^	6.56 ± 1.68^a^	72.85^a^
Overall acceptance	7.56 ± 1.42^a^	83.96^a^	7.40 ± 1.45^a^	82.25^a^

Abbreviations: H10, burger with 10% mixed fruit juice residue flour substitution; H15, burger with 15% mixed fruit juice residue flour substitution. Results are expressed as mean ± standard deviation (*n* = 98 panelists), where 1 = disliked extremely, 2 = disliked very much, 3 = disliked moderately, 4 = disliked slightly, 5 = neither liked nor disliked, 6 = liked slightly, 7 = liked moderately, 8 = liked very much, and 9 = liked extremely. Different letters within the same row indicate significant differences according to Tukey's test (*p* < 0.05).

Considering the use of a nine‐point hedonic scale, these values correspond to “liked slightly” to “liked moderately,” indicating positive consumer perception. Furthermore, both attributes presented acceptance indices above 70%, which is considered indicative of good market potential (Dutcosky 2015). These findings are comparable to those reported by Salvadori et al. (2025), who observed texture scores ranging from 5.99 for a burger with 100% pine nuts to 6.69 for a burger with 100% green banana biomass. They also align with the results of Mercês et al. (2025), who evaluated burgers made with green banana biomass and canned chickpeas, with texture scores ranging from 6.39 to 6.66, as assessed by non‐vegetarian panelists. Overall, these results indicate satisfactory acceptance for plant‐based burgers, considering that texture is often a primary sensory challenge in this product category.

When comparing the most accepted sensory attributes with the results reported by Santos et al. (2025), who analyzed burgers based on plant proteins and babassu byproducts in four different formulations, it was observed that even the formulation with the highest overall acceptance in that study (7.1) scored lower than the burgers tested in the present study. Other attributes, such as color and texture, showed acceptance levels similar to those found here, with scores ranging from 7.5 to 8.0 for color and from 6.3 to 7.5 for texture. These results align with the findings of Mercês et al. (2025), who reported flavor scores ranging from 7.32 to 7.88, assigned by vegetarian panelists familiar with plant‐based product standards.

Overall, these results indicate that the formulations containing mixed fruit juice residue flour (10% and 15%) showed high sensory acceptance, with values well above the 70% threshold commonly used to classify a product as well accepted (Dutcosky 2015). This is corroborated by the fact that only 10% of panelists indicated they would not purchase any of the formulations, highlighting the high market potential of the product.

Furthermore, it should be noted that the sensory evaluation was conducted only on formulations containing 10% and 15% mixed fruit juice residue flour, without the inclusion of a control formulation. The control was used solely as a technological reference for physicochemical and nutritional analyses. Therefore, consumer acceptance data reflect the comparative acceptance between formulations containing the upcycled ingredient at different inclusion levels, rather than comparisons against a conventional control burger. Future studies should include a control sample in sensory trials to enable direct comparisons of consumer preference against a baseline formulation.

Thus, the inclusion of an ingredient derived from agro‐industrial residues of fruit processing was well accepted in a savory food, strongly suggesting its potential application in a variety of products, such as cakes, cookies, granola, and cereal bars. Additionally, this strategy enhances fiber content and incorporates bioactive compounds, including anthocyanins and phenolics, which are associated with antioxidant activity. However, further studies are needed to evaluate the stability of these compounds and their antioxidant capacity following thermal processing.

## 4 Conclusion

The mixed juice processing residue, composed of strawberry, pineapple, and ginger, demonstrated significant potential for valorization through conversion into flour under mild drying conditions (45°C for 16 h), retaining phenolic compounds and anthocyanins while preserving antioxidant activity. The resulting flour exhibited a high dietary fiber content, predominantly insoluble, underscoring its technological and nutritional relevance as a functional food ingredient.

Incorporation of this mixed fruit juice residue flour into chickpea‐based burgers produced formulations with satisfactory technological performance and high sensory acceptance, with overall acceptance scores exceeding 80%. These results indicate that adding agro‐industrial residues can improve both structural and nutritional characteristics without negatively affecting consumer perception, supporting their use in plant‐based product development.

Beyond demonstrating technological feasibility, the study highlights the importance of valorizing food processing by‐products as a strategy to promote more sustainable and nutritionally enhanced food systems. The incorporation of fiber‐ and bioactive‐rich ingredients derived from agro‐industrial residues supports the development of plant‐based foods aligned with current consumer demands for healthier diets and resource‐efficient production. In this context, mixed juice residue flour represents a promising alternative for simultaneously reducing food waste and expanding the availability of functional ingredients for innovative food products.

## Author Contributions

**Letícia da Cunha Espinosa**: conceptualization, methodology, writing – original draft, writing – review and editing, investigation, visualization, formal analysis, data curation. **Tainara de Moraes Crizel**: methodology, investigation, data curation, writing – original draft. **Paola de Souza**: investigation. **Giovanna Ribeiro Varesano**: investigation. **Aldrey Nathália Ribeiro Corrêa**: methodology, visualization. **Cecília Roratto Köhn**: writing – review and editing. **Adriano Brandelli**: writing – review and editing, resources. **Simone Hickmann Flôres**: supervision, funding acquisition, writing – review and editing, resources, conceptualization, formal analysis. **Alessandro de Oliveira Rios**: supervision, writing – review and editing, resources, conceptualization.

## Conflicts of Interest

The authors declare no conflicts of interest.

## References

[jfds71331-bib-0001] Ajila, C. M. , K. Leelavathi , and U. J. S. Prasada Rao . 2008. “Improvement of Dietary Fiber Content and Antioxidant Properties in Soft Dough Biscuits With the Incorporation of Mango Peel Powder.” Journal of Cereal Science 48, no. 2: 319–326. 10.1016/j.jcs.2007.10.001.

[jfds71331-bib-0002] Alañón, M. E. , S. Pimentel‐Moral , D. Arráez‐Román , and A. Segura‐Carretero . 2021. “HPLC‐DAD‐Q‐ToF‐MS Profiling of Phenolic Compounds From Mango (*Mangifera indica* L.) Seed Kernel of Different Cultivars and Maturation Stages as a Preliminary Approach to Determine Functional and Nutraceutical Value.” Food Chemistry 337: 127764. 10.1016/j.foodchem.2020.127764. 32795857

[jfds71331-bib-0003] Asha, S. , M. A. Zahid , and R. Parvin . 2025. “Nutritional Index Evaluation of Plant‐Based Burger Patties Developed From Banana By‐Products: Future Meat Analogues for Consumer.” Applied Food Research 5, no. 1: 100930. 10.1016/j.afres.2025.100930.

[jfds71331-bib-0004] Association of Official Analytical Chemistry . 2005. Official Methods of Analysis. 18th ed. Association of Official Analytical Chemistry.

[jfds71331-bib-0005] Barral, B. , M. Chillet , J. Minier , M. Léchaudel , and S. Schorr‐Galindo . 2017. “Evaluating the Response to *Fusarium ananatum* Inoculation and Antifungal Activity of Phenolic Acids in Pineapple.” Fungal Biology 121, no. 12: 1045–1053. 10.1016/j.funbio.2017.09.002. 29122176

[jfds71331-bib-0006] Biazotto, K. R. , A. C. H. Xavier , R. R. de Mattos , et al. 2025. “Plant‐Based Burgers in the Spotlight: A Detailed Composition Evaluation and Comprehensive Discussion on Nutrient Adequacy.” Foods 14, no. 3: 372. 10.3390/foods14030372. 39941965 PMC11817254

[jfds71331-bib-0007] Bourne, M. C. 1990. “Basic Principles of Food Texture Measurement.” In Dough Rheology and Baked Product Texture, 331–341. Springer US. 10.1007/978-1-4613-0861-4_6.

[jfds71331-bib-0008] Campos, D. A. , T. B. Ribeiro , J. A. Teixeira , L. Pastrana , and M. M. Pintado . 2020. “Integral Valorization of Pineapple (*Ananas comosus* L.) By‐Products Through a Green Chemistry Approach Towards Added Value Ingredients.” Foods 9, no. 1: 60. 10.3390/foods9010060. 31936041 PMC7022615

[jfds71331-bib-0009] Cano‐Chauca, M. , P. C. Stringheta , A. M. Ramos , and J. Cal‐Vidal . 2005. “Effect of the Carriers on the Microstructure of Mango Powder Obtained by Spray Drying and Its Functional Characterization.” Innovative Food Science & Emerging Technologies 6, no. 4: 420–428. 10.1016/j.ifset.2005.05.003.

[jfds71331-bib-0010] CODEX ALIMENTARIUS . 1985. Codex Standard 152–1985: STANDARD FOR WHEAT FLOUR.

[jfds71331-bib-0011] Crizel, T. , M. de , A. Jablonski , A. de Oliveira Rios , R. Rech , and S. H. Flôres . 2013. “Dietary Fiber From Orange Byproducts as a Potential Fat Replacer.” LWT—Food Science and Technology 53, no. 1: 9–14. 10.1016/j.lwt.2013.02.002.

[jfds71331-bib-0012] da Cunha Espinosa, L. , A. N. R. Corrêa , A. Brandelli , S. H. Flôres , and A. de Oliveira Rios . 2026. “Physicochemical Characterization and Phenolic Compound Content of Flour From Blended Juice Residues.” Journal of the Science of Food and Agriculture 106: 4331–4340. 10.1002/jsfa.70523. 41714166 PMC13067078

[jfds71331-bib-0013] Dalal, N. , N. Phogat , V. Bisht , and U. Dhakar . 2020. “Potential of Fruit and Vegetable Waste as a Source of Pectin.” International Journal of Chemical Studies 8, no. 1: 3085–3090. https://independent.academia.edu/Bishtvinita. 10.22271/chemi.2020.v8.i1au.8739.

[jfds71331-bib-0014] Dalsasso, R. R. , G. A. Valencia , and A. R. Monteiro . 2022. “Impact of Drying and Extractions Processes on the Recovery of Gingerols and Shogaols, the Main Bioactive Compounds of Ginger.” Food Research International 154: 111043. 10.1016/j.foodres.2022.111043. 35337584

[jfds71331-bib-0015] Da Rosa Monte Machado, G. , S. Neiva Lavorato , W. Lopes , et al. 2022. “A Chloroacetamide Derivative as a Potent Candidate for Fusariosis Treatment.” Brazilian Journal of Microbiology 53, no. 3: 1289–1295. 10.1007/s42770-022-00771-9. 35648381 PMC9433475

[jfds71331-bib-0016] Da Silva, C. S. O. , R. C. P. Luna , M. G. C. A. Monteiro , et al. 2026. “Association of Cardiometabolic and Lifestyle Risk Variables With High‐Density Lipoprotein Metabolites in Hypertensive and Normotensive Overweight Women: Findings From a Randomized Trial of Mixed Dietary Fibers.” Clinical Nutrition ESPEN 71: 102846. 10.1016/j.clnesp.2025.102846. 41360166

[jfds71331-bib-0017] Da Silva, R. D. C. S. , J. A. Camponogara , C. A. A. Farias , et al. 2025. “Synergistic Effects Evaluation of Jabuticaba and Strawberry Extracts on Oxidative Stability of Pork Burgers.” Meat Science 219. 10.1016/j.meatsci.2024.109685. 39413692

[jfds71331-bib-0018] Dekkers, B. L. , R. M. Boom , and A. J. van der Goot . 2018. “Structuring Processes for Meat Analogues.” Trends in Food Science & Technology 81: 25–36. 10.1016/j.tifs.2018.08.011.

[jfds71331-bib-0019] De Marchi, M. , A. Costa , M. Pozza , A. Goi , and C. L. Manuelian . 2021. “Detailed Characterization of Plant‐Based Burgers.” Scientific Reports 11, no. 1: 2049. 10.1038/s41598-021-81684-9. 33479456 PMC7820238

[jfds71331-bib-0020] De Moraes Crizel, T. , V. S. Hermes , A. De Oliveira Rios , and S. H. Flôres . 2016. “Evaluation of Bioactive Compounds, Chemical and Technological Properties of Fruits Byproducts Powder.” Journal of Food Science and Technology 53, no. 11: 4067–4075. 10.1007/s13197-016-2413-7. 28035162 PMC5156650

[jfds71331-bib-0021] de Oliveira, I. , Y. Aquino , M. Carocho , et al. 2026. “Characterization of Newly Developed Protein Soy‐Based Burger Formulations Incorporated With Dried Unconventional Food Plants.” Food Chemistry 500: 147460. 10.1016/j.foodchem.2025.147460. 41380552

[jfds71331-bib-0022] de Souza, M. P. , F. D. de Amorim , M. R. A. Ferreira , L. A. L. Soares , and M. A. Melo . 2022. “Oxidative and Storage Stability in Beef Burgers From the Use of Bioactive Compounds From the Agro‐Industrial Residues of Passion Fruit (*Passiflora edulis*).” Food Bioscience 48: 101823. 10.1016/j.fbio.2022.101823.

[jfds71331-bib-0023] Dhalaria, R. , R. Verma , D. Kumar , et al. 2020. “Bioactive Compounds of Edible Fruits With Their Anti‐Aging Properties: A Comprehensive Review to Prolong Human Life.” Antioxidants 9, no. 11: 1–38. 10.3390/antiox9111123. PMC7698232 33202871

[jfds71331-bib-0024] Dhillon, G. K. , C. Bishnoi , and J. Kaur . 2026. “Sensory Perception of Cookies Enriched With Grapefruit Peel Powder: Bitterness as a Challenge and an Opportunity.” Journal of Culinary Science & Technology 24, no. 4: 1–16. 10.1080/15428052.2026.2635590.

[jfds71331-bib-0025] Durán‐Aranguren, D. D. , L. F. Muñoz‐Daza , L. J. Castillo‐Hurtado , et al. 2023. “Design of a Baked Good Using Food Ingredients Recovered From Agro‐Industrial By‐Products of Fruits.” LWT—Food Science and Technology 185: 115174. 10.1016/j.lwt.2023.115174.

[jfds71331-bib-0026] Dutcosky, S. D. 2015. Análise Sensorial de Alimentos. 4th ed. Curitiba: Editora Champagnat.

[jfds71331-bib-0027] De Vargas, E. F. , A. Jablonsky , S. H. Flores , and A. De Oliveira Rios . 2016. “Pelargonidin 3‐Glucoside Extraction From the Residue From Strawberry Processing (*Fragaria* × *Ananassa*).” Current Bioactive Compounds 12, no. 4: 269–275. 10.2174/1573407212666160512120242.

[jfds71331-bib-0028] Fernandes, D. C. , G. F. Dos Santos , M. O. Borges , T. Dias , and M. M. V. Naves . 2024. “Blend of Baru (*Dipteryx alata* Vog.) By‐Products as Nutritive and Healthy Food Ingredients: Chemical Composition, Functional Properties and Application in Plant‐Based Burger.” Plant Foods for Human Nutrition 79, no. 3: 578–585. 10.1007/s11130-024-01185-8. 38795267

[jfds71331-bib-0029] Fernández‐López, J. , E. Sendra‐Nadal , C. Navarro , E. Sayas , M. Viuda‐Martos , and J. A. P. Alvarez . 2009. “Storage Stability of a High Dietary Fibre Powder From Orange By‐Products.” International Journal of Food Science & Technology 44, no. 4: 748–756. 10.1111/j.1365-2621.2008.01892.x.

[jfds71331-bib-0030] Figuerola, F. , M. L. Hurtado , A. M. Estévez , I. Chiffelle , and F. Asenjo . 2005. “Fibre Concentrates From Apple Pomace and Citrus Peel as Potential Fibre Sources for Food Enrichment.” Food Chemistry 91, no. 3: 395–401. 10.1016/j.foodchem.2004.04.036.

[jfds71331-bib-0031] Fiorentini, M. , A. J. Kinchla , and A. A. Nolden . 2020. “Role of Sensory Evaluation in Consumer Acceptance of Plant‐Based Meat Analogs and Meat Extenders: A Scoping Review.” Foods 9, no. 9: 1334. 10.3390/foods9091334. 32971743 PMC7555205

[jfds71331-bib-0032] Gauthier, L. , M.‐N. Bonnin‐Verdal , G. Marchegay , et al. 2016. “Fungal Biotransformation of Chlorogenic and Caffeic Acids by *Fusarium graminearum*: New Insights in the Contribution of Phenolic Acids to Resistance to Deoxynivalenol Accumulation in Cereals.” International Journal of Food Microbiology 221: 61–68. 10.1016/j.ijfoodmicro.2016.01.005. 26812586

[jfds71331-bib-0033] Ghani, K. , T. Kausar , M. Bilal , et al. 2026. “An Updated and Comprehensive Review About Fruits and Vegetables Processing Pomace Enriched Dairy Products.” Food Production, Processing and Nutrition 8, no. 1: 7. 10.1186/s43014-025-00355-8.

[jfds71331-bib-0034] Gonçalves, L. A. , J. M. Lorenzo , and M. A. Trindade . 2021. “Fruit and Agro‐Industrial Waste Extracts as Potential Antimicrobials in Meat Products: A Brief Review.” Foods 10, no. 7: 1469. 10.3390/foods10071469. 34201960 PMC8306866

[jfds71331-bib-0035] Gorjanović, S. , D. Micić , F. Pastor , et al. 2020. “Evaluation of Apple Pomace Flour Obtained Industrially by Dehydration as a Source of Biomolecules With Antioxidant, Antidiabetic and Antiobesity Effects.” Antioxidants (Basel) 9, no. 5: 413. 10.3390/antiox9050413. 32408574 PMC7278621

[jfds71331-bib-0036] Grand View Research, Inc .. 2024. Plant‐Based Meat Market Size To Reach $24.77Bn By 2030. https://www.grandviewresearch.com/press‐release/global‐plant‐based‐meat‐market.

[jfds71331-bib-0037] Hadidi, M. , J. C. Orellana‐Palacios , F. Aghababaei , D. J. Gonzalez‐Serrano , A. Moreno , and J. M. Lorenzo . 2022. “Plant By‐Product Antioxidants: Control of Protein–Lipid Oxidation in Meat and Meat Products.” LWT—Food Science and Technology 169: 114003. 10.1016/j.lwt.2022.114003.

[jfds71331-bib-0038] Hasan, M. M. , M. R. Islam , A. R. Haque , M. R. Kabir , and S. M. K. Hasan . 2024. “Fortification of Bread With Mango Peel and Pulp as a Source of Bioactive Compounds: A Comparison With Plain Bread.” Food Chemistry Advances 5: 100783. 10.1016/j.focha.2024.100783.

[jfds71331-bib-0039] Ispiryan, A. , and E. Jarienė . 2026. “Primary Fermentation in Wine Production Influence on Phenolic Retention and Valorization Potential of Berry Skin By‐Products.” Plants 15, no. 2: 296. 10.3390/plants15020296. 41600103 PMC12845066

[jfds71331-bib-0040] Kea, T. , S. Hun , M. A. R. Mazumder , et al. 2025. “Plant‐Based Burger Patties: Effect of Chickpea and Textured Vegetable Protein Concentrations on Their Properties and In Vitro Digestibility.” Applied Food Research 5, no. 2: 101270. 10.1016/j.afres.2025.101270.

[jfds71331-bib-0041] Kong, J. , Z. Zhou , Z. Li , J. Shu , and S. Zhang . 2025. “Enriched Flavonoid Compounds Confer Enhanced Resistance to *Fusarium*‐Induced Root Rot in Oil Tea Plants.” Plant, Cell & Environment 48, no. 8: 5832–5846. 10.1111/pce.15553. 40243596

[jfds71331-bib-0042] Kulik, T. , K. Stuper‐Szablewska , K. Bilska , et al. 2017. “ *trans*‐Cinnamic and Chlorogenic Acids Affect the Secondary Metabolic Profiles and Ergosterol Biosynthesis by *Fusarium culmorum* and *F. graminearum* Sensu Stricto.” Toxins 9, no. 7: 198. 10.3390/toxins9070198. 28640190 PMC5535145

[jfds71331-bib-0043] Kyriakopoulou, K. , J. K. Keppler , and A. J. van der Goot . 2021. “Functionality of Ingredients and Additives in Plant‐Based Meat Analogues.” Foods 10, no. 3: 600. 10.3390/foods10030600. 33809143 PMC7999387

[jfds71331-bib-0044] Li, K. , C. Ma , T. Jian , et al. 2017. “Making Good Use of the Byproducts of Cultivation: Green Synthesis and Antibacterial Effects of Silver Nanoparticles Using the Leaf Extract of Blueberry.” Journal of Food Science and Technology 54, no. 11: 3569–3576. 10.1007/s13197-017-2815-1. 29051652 PMC5629166

[jfds71331-bib-0045] Li, M. , K. Koecher , L. Hansen , and M. G. Ferruzzi . 2016. “Phenolic Recovery and Bioaccessibility From Milled and Finished Whole Grain Oat Products.” Food & Function 7, no. 8: 3370–3381. 10.1039/C6FO00760K. 27406420

[jfds71331-bib-0046] Loveday, S. M. 2019. “Food Proteins: Technological, Nutritional, and Sustainability Attributes of Traditional and Emerging Proteins.” Annual Review of Food Science and Technology 10, no. 1: 311–339. 10.1146/annurev-food-032818-121128. 30649962

[jfds71331-bib-0047] Mallmann, L. P. , B. Tischer , M. Vizzotto , E. Rodrigues , and V. Manfroi . 2020. “Comprehensive Identification and Quantification of Unexploited Phenolic Compounds From Red and Yellow Araçá (*Psidium cattleianum Sabine*) by LC‐DAD‐ESI‐MS/MS.” Food Research International 131: 108978. 10.1016/j.foodres.2020.108978. 32247464

[jfds71331-bib-0048] Mao, T. , F. Huang , X. Zhu , D. Wei , and L. Chen . 2021. “Effects of Dietary Fiber on Glycemic Control and Insulin Sensitivity in Patients With Type 2 Diabetes: A Systematic Review and Meta‐Analysis.” Journal of Functional Foods 82: 104500. 10.1016/j.jff.2021.104500.

[jfds71331-bib-0049] Martínez, G. , M. Regente , S. Jacobi , M. Del Rio , M. Pinedo , and L. de la Canal . 2017. “Chlorogenic Acid Is a Fungicide Active Against Phytopathogenic Fungi.” Pesticide Biochemistry and Physiology 140: 30–35. 10.1016/j.pestbp.2017.05.012. 28755691

[jfds71331-bib-0050] Martínez, R. , P. Torres , M. A. Meneses , J. G. Figueroa , J. A. Pérez‐Álvarez , and M. Viuda‐Martos . 2012. “Chemical, Technological and In Vitro Antioxidant Properties of Mango, Guava, Pineapple and Passion Fruit Dietary Fibre Concentrate.” Food Chemistry 135, no. 3: 1520–1526. 10.1016/j.foodchem.2012.05.057. 22953888

[jfds71331-bib-0051] McClements, D. J. 2020. “Development of Next‐Generation Nutritionally Fortified Plant‐Based Milk Substitutes: Structural Design Principles.” Foods 9, no. 4: 421. 10.3390/foods9040421. 32260061 PMC7231295

[jfds71331-bib-0052] Mercês, Z. C. , N. M. Salvadori , S. M. Evangelista , et al. 2025. “Sensory Evaluation of Plant‐Based Burgers Using Green Banana Biomass, Chickpea and Teff: An Approach With Vegetarian and Non‐Vegetarian Assessors.” Food and Humanity 5: 100776. 10.1016/j.foohum.2025.100776.

[jfds71331-bib-0053] Miri, J. C. , M. B. Egea , T. L. Souza , and M. C. P. M. Lima . 2025. “Pineapple By‐Product and Green Banana Flour Evaluation and Their Application as Ingredients for Cake Formulation.” Food Science and Technology 45. 10.5327/fst.533.

[jfds71331-bib-0054] Moyer, R. A. , K. E. Hummer , C. E. Finn , B. Frei , and R. E. Wrolstad . 2002. “Anthocyanins, Phenolics, and Antioxidant Capacity in Diverse Small Fruits: Vaccinium, Rubus, and Ribes.” Journal of Agricultural and Food Chemistry 50, no. 3: 519–525. 10.1021/jf011062r. 11804523

[jfds71331-bib-0055] Nasrollahzadeh, F. , N. Alexi , K. B. Skov , L. Roman , K. Sfyra , and M. M. Martinez . 2024. “Texture Profiling of Muscle Meat Benchmarks and Plant‐Based Analogues: An Instrumental and Sensory Design Approach With Focus on Correlations.” Food Hydrocolloids 151: 109829. 10.1016/j.foodhyd.2024.109829.

[jfds71331-bib-0056] Nemzer, B. , L. Vargas , X. Xia , M. Sintara , and H. Feng . 2018. “Phytochemical and Physical Properties of Blueberries, Tart Cherries, Strawberries, and Cranberries as Affected by Different Drying Methods.” Food Chemistry 262: 242–250. 10.1016/j.foodchem.2018.04.047. 29751916

[jfds71331-bib-0057] Nogueira, T. B. B. , L. S. Silva , C. F. A. Lima , et al. 2025. “Transforming Waste Into a Bioactive Resource: Phenolic Profile, Antioxidant, and Antimutagenic Properties of Pineapple (Ananas comosus) Crown Infusion.” Sustainable Food Technology 4, no. 1: 1115–1125. 10.1039/d5fb00445d.

[jfds71331-bib-0058] Pereira, R. , C. Velasco , R. Gómez‐Garcia , J. Dias , M. Pintado , and L. M. P. Valente . 2024. “Unravelling the Effects of Extrusion and Drying Temperatures on the Radical Scavenging Capacity of Aquafeeds Supplemented With Mango and Pineapple By‐Products.” Animal Feed Science and Technology 316: 116061. 10.1016/j.anifeedsci.2024.116061.

[jfds71331-bib-0059] Portincasa, P. , L. Bonfrate , M. Vacca , et al. 2022. “Gut Microbiota and Short Chain Fatty Acids: Implications in Glucose Homeostasis.” International Journal of Molecular Sciences 23, no. 3: 1105. 10.3390/ijms23031105. 35163038 PMC8835596

[jfds71331-bib-0060] Pukalskienė, M. , A. Pukalskas , L. Dienaitė , et al. 2021. “Recovery of Bioactive Compounds From Strawberry (*Fragaria* × *Ananassa*) Pomace by Conventional and Pressurized Liquid Extraction and Assessment Their Bioactivity in Human Cell Cultures.” Foods 10, no. 8: 1780. 10.3390/foods10081780. 34441558 PMC8392826

[jfds71331-bib-0061] Rabetafika, H. N. , B. Bchir , M. Aguedo , M. Paquot , and C. Blecker . 2014. “Effects of Processing on the Compositions and Physicochemical Properties of Fibre Concentrate From Cooked Fruit Pomaces.” Food and Bioprocess Technology 7, no. 3: 749–760. 10.1007/s11947-013-1073-0.

[jfds71331-bib-0062] Rangani, S. C. , and K. K. D. S. Ranaweera . 2023. “Incorporation of Natural Antioxidants Extracted From Strawberry, Cinnamon, Beetroot, and Ginger; Into Virgin Coconut Oil for Expansion of Its Shelf Life.” Applied Food Research 3, no. 2: 100325. 10.1016/j.afres.2023.100325.

[jfds71331-bib-0063] Research and Markets . 2026. Plant Based Food Market Size, Competitors & Forecast to 2030. Market Report. Dublin: Research and Markets.

[jfds71331-bib-0064] Rizzello, C. G. , M. Verni , S. Bordignon , V. Gramaglia , and M. Gobbetti . 2017. “Hydrolysate From a Mixture of Legume Flours With Antifungal Activity as an Ingredient for Prolonging the Shelf‐Life of Wheat Bread.” Food Microbiology 64: 72–82. 10.1016/j.fm.2016.12.003. 28213038

[jfds71331-bib-0065] Russo, R. , M. A. Costa , N. Lampiasi , et al. 2023. “A New Ginger Extract Characterization: Immunomodulatory, Antioxidant Effects and Differential Gene Expression.” Food Bioscience 53: 102746. 10.1016/j.fbio.2023.102746.

[jfds71331-bib-0066] Salvadori, N. M. , Z. da Conceição das Mercês , S. Evangelista , et al. 2025. “Physico‐Chemical and Sensory Quality of Plant‐Based Burgers Made With Pine Nuts (*Araucaria angustifolia*) and Green Banana (*Musa* spp.) Biomass.” International Journal of Gastronomy and Food Science 41: 101254. 10.1016/j.ijgfs.2025.101254.

[jfds71331-bib-0067] Santos, D. , J. A. Lopes da Silva , and M. Pintado . 2022. “Fruit and Vegetable By‐Products' Flours as Ingredients: A Review on Production Process, Health Benefits and Technological Functionalities.” LWT—Food Science and Technology 154: 112707. 10.1016/j.lwt.2021.112707.

[jfds71331-bib-0068] Santos, V. S. A. , P. H. M. Sousa , G. M. V. C. Nunes , and S. D. Benevides . 2025. “Perfil Sensorial de Análogo de Hambúrguer Vegetal À Base de Coprodutos de Babaçu e Proteínas Vegetais.” Nutrivisa Revista de Nutrição e Vigilância Em Saúde 12, no. 1: e14748. 10.52521/nutrivisa.v12i1.14748.

[jfds71331-bib-0069] Šarić, B. , T. Dapčević‐Hadnađev , M. Hadnađev , et al. 2019. “Fiber Concentrates From Raspberry and Blueberry Pomace in Gluten‐Free Cookie Formulation: Effect on Dough Rheology and Cookie Baking Properties.” Journal of Texture Studies 50, no. 2: 124–130. 10.1111/jtxs.12374. 30345519

[jfds71331-bib-0070] Sha, L. , and Y. L. Xiong . 2020. “Plant Protein‐Based Alternatives of Reconstructed Meat: Science, Technology, and Challenges.” Trends in Food Science & Technology 102: 51–61. 10.1016/j.tifs.2020.05.022.

[jfds71331-bib-0071] Statista . “Global Fruit Production Volume in 2023, by Selected Variety.” 2025. Accessed March 18, 2026. https://www.statista.com/statistics/264001/worldwide‐production‐of‐fruit‐by‐variety/.

[jfds71331-bib-0072] Tamkutė, L. , G. Jančiukė , M. Pukalskienė , I. Sarapinienė , V. Arvydas Skeberdis , and P. Rimantas Venskutonis . 2022. “Cranberry and Black Chokeberry Extracts Isolated With Pressurized Ethanol From Defatted by Supercritical CO_2_ Pomace Inhibit Colorectal Carcinoma Cells and Increase Global Antioxidant Response of Meat Products During In Vitro Digestion.” Food Research International 161: 111803. 10.1016/j.foodres.2022.111803. 36192948

[jfds71331-bib-0073] Vargas, V. , S. Saldarriaga , F. S. Sánchez , L. N. Cuellar , and G. M. Paladines . 2024. “Effects of the Spray‐Drying Process Using Maltodextrin on Bioactive Compounds and Antioxidant Activity of the Pulp of the Tropical Fruit Açai (*Euterpe oleracea* Mart.).” Heliyon 10, no. 13: e33544. 10.1016/j.heliyon.2024.e33544. 39040403 PMC11260920

[jfds71331-bib-0074] Viuda‐Martos, M. , E. Sánchez‐Zapata , A. Martin‐Sánchez , et al. 2012. “Technological Properties of Pomegranate (*Punica granatum* L.) Peel Extract Obtained as Co‐Product in the Juice Processing.” In Dietary Fiber and Health, edited by N. Almeida S. Cho , and S. Cho , 539. Taylor & Francis. https://books.google.com.br/books?hl=pt‐BR&lr=&id=TJdZI5WZ8k0C&oi=fnd&pg=PA443&dq=Technological+properties+of+pomegranate+(Punica+granatum+L.)+peel+extract+obtained+as+co‐product+in+the+juice+processing.&ots=A‐rtZnSbiW&sig=tip9lO82Nj_‐SJr3yZiIBl1o8NM.

[jfds71331-bib-0075] von Muhlenbrock, C. , F. Aronsohn , R. Quera , and A. M. Madrid . 2025. “The Role of Dietary Fiber in the Gastrointestinal Tract: When, How and Why?” Best Practice & Research Clinical Gastroenterology 79: 102080. 10.1016/j.bpg.2025.102080. 41423309

[jfds71331-bib-0076] Wallace, T. C. , and M. M. Giusti . 2015. “Anthocyanins.” Advances in Nutrition 6, no. 5: 620–622. 10.3945/an.115.009233. 26374184 PMC4561837

[jfds71331-bib-0077] Zamani, S. , O. S. Agu , and V. Meda . 2026. “Valorization of Fruit and Vegetable Waste and By‐Products Into Bioactive‐Rich Powders for Functional Food Applications: A Review.” Food and Bioprocess Technology 19, no. 5: 213. 10.1007/s11947-026-04277-2.

[jfds71331-bib-0078] Zayas, J. F. 1997. “Water Holding Capacity of Proteins.” In Functionality of Proteins in Food, 76–133. Springer Berlin Heidelberg. 10.1007/978-3-642-59116-7_3.

